# Fluoride concentrations in the pineal gland, brain and bone of goosander (*Mergus merganser)* and its prey in Odra River estuary in Poland

**DOI:** 10.1007/s10653-014-9615-6

**Published:** 2014-04-18

**Authors:** Elzbieta Kalisinska, Irena Bosiacka-Baranowska, Natalia Lanocha, Danuta Kosik-Bogacka, Katarzyna Krolaczyk, Aleksandra Wilk, Katarzyna Kavetska, Halina Budis, Izabela Gutowska, Dariusz Chlubek

**Affiliations:** 1 Department of Biology and Medical Parasitology, Pomeranian Medical University, Powstancow Wielkopolskich 72, 70-111 Szczecin, Poland; 2 Department of Biochemistry and Medical Chemistry, Pomeranian Medical University, Powstancow Wielkopolskich 72, 70-111 Szczecin, Poland; 3 Laboratory of Biology and Ecology of Parasites, West Pomeranian University of Technology, Doktora Judyma 10, 71-466 Szczecin, Poland; 4 Department of Biochemistry and Human Nutrition, Pomeranian Medical University, Szczecin, Poland

**Keywords:** Fluoride concentration, Brain, Pineal gland, Bone, Bird, Fish

## Abstract

The aim of the study was to investigate fluoride concentrations in bone, brain and pineal gland of goosander *Mergus merganser* wintering in the Odra estuary (Poland) as well as in fish originating from its digestive tract. The fluoride concentrations were determined with potentiometric method. Medians of concentrations in goosander had the highest and the lowest values in pineal gland and brain (>760 and <190 mg/kg, respectively). Fluoride concentration in the pineal gland was significantly greater than in the bone and the brain of the duck. In fish, the fluoride concentration ranged from 37 to 640 mg/kg and significant correlation was revealed between the fluoride concentration and fish weight and length. Based on own results and data of other authors, a daily fluoride intake by the goosander in the Odra estuary was estimated at 15 mg. So high fluoride concentrations like in the duck have not been found in mammal brains.

## Introduction

Humans and animals are exposed to fluoride mainly via water, food and air. The natural fluoride concentration varies strongly and depends on the local geological structure and also on local/regional human activity. In many regions of the world, including Europe, F^−^ in ground and surface water sometimes exceeds 1–1.5 mg/l, the level considered to be safe for humans (Fawell et al. 2006; Fordyce et al. 2007; Berger et al. 2012; Ghosh et al. 2013).

Fluoride content in plants and animal food depends on its concentration in soil, water used for irrigation and air deposition. Diet consisting of fish and aquatic invertebrates is especially rich in F^−^, albeit freshwater fish usually show higher F^−^ levels than marine species (Kay et al. 1975; WHO 2000; Camargo 2003). In ecotoxicological research on vertebrates, F^−^ is usually determined in teeth and antlers. In the hard tissues, F^−^ reacts with crystals of hydroxyapatite (calcium hydroxyphosphate), thus forming a mineral scaffolding for bones. Vertebrates reveal different effects of exposure to F^−^, depending on the F^−^ concentration and duration of exposure. Micromolar F^−^ concentrations promote cell proliferation and growth, whereas millimolar concentrations suppress cell proliferation and induct apoptosis in hard tissues (Teotia et al. 2004; Agalakova and Gusev 2012).

Due to the high affinity of F^−^ to strongly mineralized tissues, F^−^ concentrations reach the highest levels in the bones and teeth, and so these materials are frequently used in the bioindication of long-term exposure to F^−^ (Kay et al. 1975; Bezerra de Menezes et al. 2003; Kierdorf et al. 2012; Ghosh et al. 2013). Excessive supply of F^−^ in water and diet results in dental and skeletal fluorosis among people and wild and domesticated animals (Greenwood et al. 1946; Machoy et al. 1995; Suttie et al. 1987; Walton 1988; Kierdorf et al. 1995; Bezerra de Menezes et al. 2003; Gutowska et al. 2004; Teotia et al. 2004; Zemek et al. 2006; Richter et al. 2010; Kalisinska and Palczewska-Komsa 2011; Choubisa 2012). Compared with numerous studies on F^−^ in the bone of mammals, the number of analogous works on birds is low (Bird et al. 1989; Henny and Burke 1990; Robertson and Lock 1994; Vikoren and Stuve 1995; Xie and Sun 2003).

Intensely developing in vivo and in vitro research provides a growing body of evidence pointing to the negative effects of excessive F^−^ on mammals, from the subcellular to the ecological levels (Walton 1988; Shore 1995; Barbier et al. 2010). One of the most important areas of research is the evaluation of the effect of F^−^ on the nervous system and endocrine glands of mammals, including the pineal gland (Varner et al. 1998; Choi et al. 2012). It has been shown experimentally that F^−^ has neurotoxic properties and adversely affects the functioning of the brain, even at small doses. It contributes to the induction of apoptosis of neurons, formation of oxidative stress, increased amounts of free radicals and lipid peroxidation in the brain and inhibits the production of antioxidant enzymes, mitochondrial enzymes of energy and glutamate transporters. The result is a decreased activity in the mouse and rat brain, impaired memory and learning ability in animals and humans (Blaylock 2004; Bharti and Srivastava 2009; Reddy et al. 2009; Barbier et al. 2010; Basha and Sujitha 2012; Choi et al. 2012).

Although the influence of F^−^ on nervous tissues has been intensely studied for decades, the number of publications on F^−^ concentrations in the brain is low and they are almost exclusively carried out on humans and laboratory mammals (Mayer and Gross 1985; Tsunoda et al. 2005; Martinez et al. 2007; Reddy et al. 2011; Lubkowska et al. 2012). Although the brain is classified as a soft organ, some of its regions include highly mineralized structures. Humans and other mammals develop intracranial calcifications with age, known as acervuli (also concretions, corpora arenacea or brain sands), a normal physiological phenomenon in the brain, especially in the pineal gland (Makariou and Patsalides 2009; Uduma et al. 2011). In mammals, the pineal acervuli have long been studied with regard to their association with aging, melatonin production or neurological disorders (Arendt 1998; Baconnier et al. 2002; Kim et al. 2012). In contrast, in birds, the occurrence of intracranial calcifications is very poorly understood. So far acervuli have been found in the pineal gland of the goose, duck and turkey (Fejer et al. 2001; Przybylska-Gornowicz et al. 2009). Research by Luke (2001) showed that in the human pineal gland F^−^ reaches very high concentrations, greater than in the bones, which is probably related to the presence of fluorapatite-containing concretions in the gland. We have not found any other papers regarding F^−^ in the mammal and avian pineal gland in available literature. The only exception is one conference report (Kalisinska et al. 2012).

Compared with mammals, the isolation of the pineal gland of birds is much simpler due to easier access. Although the pineal gland is part of the epithalamus, in birds, including ducks, it is clearly visible from the dorsal side of the brain. This elongated gland is located between the cerebellum and the cerebral hemispheres. Its distal part is fused with the dura mater adjacent to the skull, and the anterior part is connected to the area of commissure of the epithalamus. In Anseriformes, including ducks, the pineal gland is relatively large with a length from 5 to 12 mm (Lewczuk et al. 2000; Prusik et al. 2006).

The aim of this study was to investigation F^−^ concentrations in the skull bone, brain and pineal gland of the goosander *Mergus merganser*, a piscivorous duck wintering in northwestern Poland, as well as in fish collected from its esophagus and stomach.

## Methods

### Study area

Szczecin is the largest city in the Odra Delta estuary (410,000 inhabitants). The western and eastern Odra Rivers flow through the city, and the industrialized northern part of Miedzyodrze and the shallow and extensive (about 54 km^2^) Lake Dabie are also within the city limits. Szczecin used to be a significant industrial center, with shipyards, power plants, “Fosfan” phosphate fertilizer factory, and, until recently, a paper mill and chemical plants. Near Szczecin, on the Odra River, there are two such large plants, “Police” (a major producer of phosphatic fertilizers) and a conventional coal power station “Elektrownia Dolna Odra” (Lower Odra River Power Plant), in operation since 1969 and 1974, respectively, and in the past, the major sources of environmental pollution with fluoride. Gradual upgrades have significantly reduced their emissions, but the heaps of phosphogypsum and ash, the main by-products, are still a significant problem (Straszko et al. 1996; Piekos and Paslawska 1998). The waters of the Odra Delta, especially in the Szczecin Lagoon, are significantly contaminated, particularly with trace metals (Glasby et al. 2004; HELCOM 2004). Yet the effects of these pollutants on birds and their food items are still poorly known (Kalisinska et al. 2010, 2012).

Individuals of goosander were gathered during the second half of January 2010. The collected birds came from one of the largest Miedzyodrze canals named Parnica (53° 24′ 44.60″ N and 14° 33′15.92″ E), which connects Lake Dabie and the western Odra. A small goosander flock was accidentally trapped by stationary fishing nets deployed in a canal near the Lake Dabie and drowned.

### Sampling

A laboratory examination determined the body weight (to an accuracy of 5 g), sex and age category (immature or adult) of 45 goosanders. Sex determination was also based on the morphology of gonads. The most certain way of differentiating 1 year old and older ducks is the inspection of the bursa of Fabricius, a lymphatic gland, which is the largest in first-year birds and atrophies with age and maturation (Siegel-Causey 1990). Before chemical analysis, 32 goosander heads were stored in labeled plastic bags at −20 °C. From the partially thawed heads, the top skull bones, the brain and isolated pineal gland were taken.

Eleven (11) fish of unspecified species were found in the digestive tract of eight goosanders (in the esophagus and/or stomach). The fish were weighed and measured. Not all fish were complete (two were without head, one without head and tail fin, one without a tail fin). The goosander samples and whole fish were dried to constant weight at 55° C. The water content in the samples was determined by gravimetric method.

### Fluoride concentration measurement

Whole goosander pineal glands and 10 mg of each pulverized bone, brain and fish samples were used in the analytical procedure. Samples were dissolved in 0.1 M of perchloric acid and mixed for 1 h at 95° C. Fluoride concentration was determined by potentiometric method using an Orion ion-selective electrode (Gutowska et al. 2009). Fluoride concentrations are presented for dry weight (dw).

### Statistical analysis

A Student’s *t* test was used to evaluate the differences between the mean weights of the body and the brain in the analyzed groups of goosanders. The conformity of the distribution of F^−^ concentrations in goosander samples with an expected normal distribution was examined by Kolmogorov–Smirnov test (KS test), with a Lilliefors correction. Because a lack of conformity was observed for the bone and pineal gland samples, further statistical procedures were based on a nonparametric Mann–Whitney test. Comparisons of the mean F^−^ were carried out between the two groups of samples distinguished by gender, age and type of biological material. We also calculated the Spearman’s rank correlation for the relationship between F^−^ concentration in various biological samples and bird age categories.

## Results

The analyzed birds included 21 adults (10 F, females, and 11 M, males) and 11 immature individuals (3 F and 8 M).

Body and brain masses of goosander ranged from 1,440 to 2,385 g and from 4.74 to 7.32 g, respectively, with mean levels at 1,875 and 6.14 g, respectively (Table 1). There were differences in body and brain weights between females and males, both in adult (ad) and immature (im) individuals, with females being significantly lighter than males in both age groups (1,610 g ad F vs 2,085 g ad M, and 1,555 im F vs 2,000 g im M). Females also had smaller brains. The weight and length of the fish ranged from 3.3–25.1 g and 70–125 mm (means 8.7 g and 90 mm).

**Table 1:** Goosander groups (Ad, adult; Im, immature; M, male; F, female), body and brain masses (g) and F^−^ concentrations (mg/kg dw) in biological samples of investigated groups

Goosander group		Body mass	Brain mass	Fluoride concentration		
				Skull bone	Brain	Pineal gland
Ad F	n	10	10	10	10	10
	Median	1,610	5.97	402.7	146.8	859.2
	AM ± SD	1,610 ± 105	5.86 ± 0.46	512.1 ± 363.9	158.4 ± 55.5	1,045.5 ± 622.0
	Range	1,440–1,775	4.94–6.50	130.2–1,158.3	86.6–257.8	456.9–2,513.0
Im F	n	3	3	3	3	3
	Median	1,555	5.08	321.1	170.7	700.9
	AM ± SD	1,546.7 ± 82.8	5.13 ± 0.42	330.0 ± 120.9	182.0 ± 24.8	722.4 ± 196.4
	Range	1,460–1,625	4.74–5.57	213.8–455.1	164.8–210.5	537.7–928.6
Ad M	n	11	11	11	11	11
	Median	2,085	6.43	657.4	151.4	727.0
	AM ± SD	2,095 ± 195	6.46 ± 0.46	666.3 ± 512.6	157.0 ± 29.3	1,086.1 ± 1,008.8
	Range	1,680–2,385	5.77–7.32	162.3–1,935.0	115.4–224.7	244.3–3,938.5
Im M	n	8	8	8	8	8
	Median	2,000	6.42	189.1	192.0	848.1
	AM ± SD	2,300 ± 180	6.43 ± 0.35	191.6 ± 44.9	186.5 ± 50.0	772.0 ± 316.1
	Range	1,795–2,320	5.91–7.00	128.0–268.9	101.6–250.4	272.4–1,134.5
Ad (F + M)	n	21	21	21	21	21
	Median	1,775	6.30	429.7	149.7	760.5
	AM ± SD	1,860 ± 290	6.18 ± 0.54	592.8 ± 444.1	158.0 ± 42.7	1,068.2 ± 826.6
	Range	1,440–2,385	4.94–7.32	130.2–1,935.0	86.6–257.8	244.3–3,938.5
Im (F + M)	n	11	11	11	11	11
	Median	1,930	6.29	201.5	188.7	813.7
	AM ± SD	1,895 ± 270	6.07 ± 0.70	229.3 ± 92.3	185.3 ± 43.4	758.5 ± 279.6
	Range	1,460–2,320	4.74–7.00	128.0–455.1	101.6–250.4	272.4–1,134.5
Ad + Im	n	32	32	32	32	32
	Median	1,880	6.29	295.0	160.7	787.1
	AM ± SD	1,875 ± 280	6.14 ± 0.59	467.9 ± 400.9	167.1 ± 44.2	961.7 ± 698.9
	Range	1,440–2,385	4.74–7.32	128.0–1,935	86.6–257.8	244.3–3,938.5

Water content of the goosander skull bone and brain as well as fish samples was 38.1, 78.6 and 75.3 %, respectively.

**Fig. 1:**
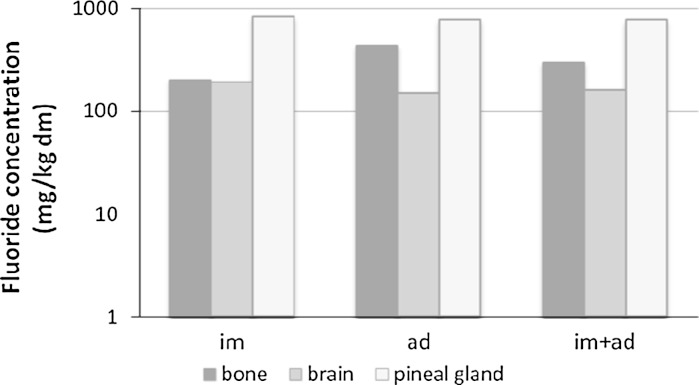
Medians of F^−^ concentration in various biological samples of immature (im), adult (ad) and all investigated goosanders from NW Poland

There were no statistically significant differences in F^−^ concentrations in bone, brain and pineal gland between F and M in any age group, so further comparisons were conducted between all adult (F + M) and immature specimens (F + M). The greatest variation in F^−^ was observed in the bones and pineal gland, while the lowest in the brain, both among adults and immature goosanders (Table 1). Median F^−^ in the tested materials from the ad and im goosanders was the highest in the pineal gland and exceeded 780 mg/kg dw, while the lowest was in the brain (<170 mg/kg dw). In the ad group, bone F^−^ was two times higher than in the im group (~430 vs ~200 mg/kg dw), but in the brain and pineal gland, it was lower than in the im group (Fig. 1). The significance of differences in F^−^ concentrations between the ad and im groups was confirmed for the bone (*p* < 0.01) and brain (*p* < 0.05).

Comparisons of F^−^ concentrations between the types of samples obtained from the ad and the im goosanders showed that in both age groups, pineal gland F^−^ was significantly higher than in the brain (*p* < 0.001) and bone (*p* < 0.01). In the goosander pineal gland, F^−^ was 4–5 times higher than in the brain. In addition, bone F^−^ was higher than in the brain in the ad group (*p* < 0.001). When taking into account all the individuals (ad + im), these differences were significant between each pair of the three compared types of samples (*p* < 0.001). In the entire group of goosanders (*n* = 32), a statistically significant value of Spearman’s correlation rank was observed between age and brain F^−^, as well as between age and bone F^−^ (*r* = −0.363 *p* < 0.05, and *r* = 0.502 *p* < 0.01, respectively).

F^−^ in the fish ranged from 37.3 to 640 mg/kg dw, with a median of 140 mg/kg dw. In 6 out of 11 examined fish specimens (55 %), F^−^ was >150 mg/kg dw. Although the species of fish were not determined, we decided to see if there was a correlation between F^−^ in the body of the fish and their weight and length. In both cases, there were significant correlations (*p* < 0.05): *r* = 0.741 and 0.660, respectively.

## Discussion

In the available literature, we found little data on F^−^ concentration in the brain and pineal gland of warm-blooded vertebrates and their relation to bone F^−^ concentrations. Our research on birds, similar to Luke (2001) who examined the human pineal gland and bone showed that F^−^ concentration in the gland was much higher than in bone.

### Bone F^−^ in birds

In farm ducks (*Anas platyrhynchos*), 99.5 % of F^−^ is stored in the skeleton (28.7 % in the skull and 69.8 % in other bones). In birds, F^−^ concentration is usually determined in the femur. Its concentration in the bones of wild birds is characterized by very high inter-individual and inter-species variability. In extreme cases (with penguins fed on krill), F^−^ in adults may range from 6,000 to 11,000 mg/kg dw (Culik 1987; Xie and Sun 2003).

It seems that birds can tolerate much higher F^−^ concentrations than mammals. In experimentally, F^−^-intoxicated American kestrels (*Falco sparverius*), even very high doses of NaF (sodium fluoride) in their diet (1,120 and 2,240 ppm) did not disrupt the development of internal organs, including the brain, although they did contribute to a decrease in bone breaking strength (Bird et al. 1992). In laying hens and turkeys fed with feed containing 400 mg/kg dw, no clinical interferences with normal performance were observed (Weinstein and Davison 2004). However, fluoride added to water had a significant negative effect on egg production in laying farm hens and hatching success in experimental groups of the screech-owl *Otus asio*, which received 200 mg/kg in the diet (Pattee et al. 1988; Coetzee et al. 2000).

Most data on F^−^ concentration in the bones of free-living birds can be found in the works of Stewart et al. (1974) and Robertson and Lock (1994). The latter contains data of more than 120 New Zealand species. However, these concentrations are expressed in conversions for ash, which makes it difficult to compare their results with other publications, where F^−^ concentrations are usually presented for dw. The ash content in dry avian bone mass is a variable which depends on the age of individuals, bird systematic position and environmental factors, including the supply of F^−^ in the diet (Chan et al. 1973; Bird et al. 1992; Lundy et al. 1992; Shim et al. 2011).

The share of ash increases with age, as observed in wild black-crowned night heron *Nycticorax nycticorax* and wood pigeon *Columba palumbus*. In individuals in the first year of life, it can be ~22 % while in older specimens 40–46 % (Henny and Burke 1990; Salicki and Kalisinska 2006). Shim et al. (2011) estimate that in chick bone the share of ash is 28–30 %, depending on the breed, while in the chicks of the Japanese farm quail *Coturnix japonica*, the content of ash rose to 53 % after 20 days of receiving 1.2 % Ca and 0.075 % F^−^ in their diet (Chan et al. 1973). In captive chicks of the American kestrel *F. sparverius*, experimental intoxication with NaF for 3 weeks (1,120 and 2,240 ppm) increased bone ash content to 56–57 % compared with the 52 % in the control group (Bird et al. 1992). Taking into account, the aforementioned data, it can be assumed that on average ash accounts for 45 % of the dry mass of avian bones.

Due to increasing anthropogenic pollution, it is currently very difficult to identify areas not contaminated by F^−^ and to specify F^−^ in avian bones that reflect a natural geochemical background, especially since most of these birds are migratory animals, living in various environments and consuming different types of food. It is generally accepted that in the bones of humans, cattle, swine, chicks and other birds (except penguins) from uncontaminated areas, F^−^ concentrations are between 500 and 1,000 mg/kg dw (Xie and Sun 2003). Stewart et al. (1974) suggested that natural or background F^−^ levels in avian bones coming from birds inhabiting non-industrialized area in New Zealand averaged 500–600 mg/kg dw.

Medians and arithmetic means calculated for the bone of all goosanders in this study did not exceed 300 and 470 mg/kg dw, respectively, and were lower than the values from other reports. Most goosanders wintering in the Odra Delta have their breeding grounds in northern Europe, especially in sparsely populated Nordic regions with a negligible fluoride pollution. Given that F^−^ is most intensely absorbed and most efficiently incorporated into bone during the rapid growth phases of birds and mammals in the postnatal period, it can be assumed that, to a large extent, F^−^ concentrations in goosander bones reflect the geochemical background of Scandinavia.

### Diet and F^−^

Links between F^−^ concentrations in avian bone and the type of diet were reported by Stewart et al. (1974) in birds coming from a non-contaminated area of New Zealand. The smallest concentrations of bone ash (489 mg/kg) were found in the pukeko *Porphyrio melanotus*, which feeds on plant material (stems, leaves, seed heads) and insects, and the greatest concentrations (~4,000 mg/kg) in the red-billed gull *Larus novaehollandiae*, a consumer of marine euphausiid crustaceans (rich in F^−^), small fish, beach scavenging and invertebrates coming from agricultural lands.

Studies conducted in the 1970s in North American areas distant from industrial centers showed that in Galliformes (feeding on plant food mixed with soil invertebrates), F^−^ concentrations were 100–320 mg/kg dw, while in the omnivorous magpie *Pica pica*, they were significantly higher (>530 mg/kg dw), as in the American kestrel (503–638 mg/kg dw), feeding on insects and small mammals (Kay et al. 1975; Bird et al. 1989). In comparison with these birds, in the fish-eating female and male Norwegian herring gull *Larus argentatus* from polluted (poll) and reference (ref) areas, F^−^ concentrations were higher (F ref 3,107, M ref 3,191 mg/kg ash or 1,398 and 1,436 mg/kg dw; F poll 3,615, M poll 2,512 mg/kg ash or 1,627 and 1,130 mg/kg dw, respectively).

These levels seem to be very close to those reported by Henny and Burke (1990) for two groups of piscivorous black-crowned night herons (in the second and third years of life) living adjacent to a phosphate processing complex in Idaho, USA: 2,833 and 3,760 mg/kg ash (about 1,275 and 1,690 mg/kg dw). Among New Zealand birds with fish in their diet (adult or combined age groups), which were related to coastal and inland waters, like the white-faced heron *Ardea novaehollandiae* and various cormorant species, mean F^−^ concentrations ranged from 1,490 to 3,510 mg/kg ash or about 670 to 1,580 mg/kg dw (Robertson and Lock 1994). In comparison with data from Henny and Burke (1990) as well as Robertson and Lock (1994), adult and immature goosanders wintering in the Odra delta had low mean F^−^ concentrations in skull bone (<430 and <205 mg/kg dw, respectively). In European wintering and breeding areas, adult goosander on average eat from 310 do 500 g fish a day, fish prey being usually from 50 to 170 mm long (Svenning et al. 2005; Zydelis and Kontautas 2008). The body length of most fish found in the upper digestive tract of the studied goosander from Poland was in this range. Average weight of fish prey was about 10 g and was approximately three times lower than the reported prey weight for goosander wintering at the Curonian Lagoon, in the southeast Baltic Sea (Zydelis and Kontautas 2008). It can be conservatively estimated that a ~2 kg heavy goosander wintering in the Odra Delta eats about 350 g of fish per day containing an average F^−^ concentration of 45 mg/kg wet matter (corresponding to 183 mg/kg dw). Therefore, the daily intake is considerable and amounts to ~15 mg F^−^.

In freshwater and marine fish muscle, fluoride concentrations range from 0.6 to 26 mg/kg ww and in whole fish from 10 to 60 mg/kg ww (Lall 1994; Camargo 2003).

Fish found in the digestive tract of the goosander did not deviate from the specified F^−^ concentration range. However, in fish-eating birds, stomach pH is low and periodically may be 0.9, which favors the dissolution of the bones and scales of fish (Zijlstra and Van Eerden 1995). This can result in increased availability of F^−^ and thus increased absorption in the gastrointestinal tract. It is quite possible that the blood stream and soft tissues of piscivorous goosander wintering in the Odra Delta absorbed a considerable amount of F^−^, yet accumulation in the bones was rather low and lasted no longer than the 3–4 months spent in the wintering grounds.

Mean F^−^ concentrations of the studied ad and im goosanders were much lower or similar to the respective age groups of the sedentary and plant-eating wood pigeon from the vicinity of Szczecin investigated in 2002–2004. Mean values of F^−^ levels ranged from 730 to 880 and from 200 to 390 mg/kg dw in ad and im pigeons, respectively (Salicki and Kalisinska 2006).

### Age and bone F^−^

In most wild birds (individuals mature enough to fly) age cannot be precisely determined, but usually can be qualified as immature (individuals in the first year of life) or adult (older birds), based on differences in plumage and/or stages of development of the bursa of Fabricius. Wood pigeons and goosanders from NW Poland showed a significant difference in bone F^−^ concentrations between these two age group; in both species, F^−^ was higher in the ad group (Salicki and Kalisinska 2006; also in this study).

In addition to the black-crowned night heron divided into four age groups based on differences in plumage (hatching year, second year, third year, adult: four and more years), a clear tendency of increasing accumulation of F^−^ in bone was shown with age (Henny and Burke 1990). However, it should be noted that in the chicks of some species, bone F^−^ concentrations reached very high levels (close to those recorded in older animals) over a short term, as early as during the first 2–7 weeks of life. Later, in older chicks and adults, F^−^ concentrations grow more slowly.

In the free-living Adelie penguin *Pygoscelis adeliae*, mainly feeding on krill (containing >1,000 mg/kg dw), in the femur of 2 week old chicks, F^−^ concentration was as much as about 8,000 mg/kg dw, and in 5-week-old chicks, it had risen to 9,500 mg/kg dw and later remained at a similar level (Culik 1987). Also in chickens (fed with starter and finisher feedstuff containing 180 and 110 mg/kg dw, respectively), F^−^ levels grew intensely for 30–40 days in the compact and spongy bone of the femur reaching 336 and 224 mg/kg, respectively. Later, at the 50th day of their life, F^−^ in the chicken femur had only slightly increased (Dolegowska et al. 2003).

The small number of studies on bone F^−^ in wild birds and the lack of long-term experimental observations (5+ years long) that take into account age group comparisons make it difficult to argue that F^−^ levels continue to grow in older sexually mature birds (>5 years). Among free-living birds, an age-related increase in F^−^ concentration has been documented in studies concerning immature and reproducing adult individuals of the black-crowned night heron, wood pigeon and goosander (Henny and Burke 1990; Salicki and Kalisinska 2006; this study). The respective maximum life spans of these species are 21, 17 and 14 years, although in the wild, average life expectancy does not exceed 4–5 years (http://genomics.senescence.info). Merkley (1981) suggested that once bone F^−^ levels in birds reach a certain level, they remain relatively constant, even during egg laying when Ca and F^−^ become more mobile.

### Sex and bone F^−^

There are few papers on captive or free-living birds with regard to gender differences in bone F^−^ concentrations. An experimental study carried out on screech owls in the breeding season with diets containing 200 mg/kg dw showed that females had higher residues of F^−^ in bone than males (Pattee et al. 1988). Female herring gulls acquired during the breeding season from a breeding colony located near an aluminum smelter in Karmoy (Norway) had a significantly higher bone F^−^ concentration than males (3,615 vs 2,512 mg/kg bone ash), while a difference between the sexes was not observed in the reference area (Vikoren and Stuve 1996). In the non-breeding season, in wood pigeon and goosander from an area near the city of Szczecin, gender-related differences in bone F^−^ concentration were not detected (Salicki and Kalisinska 2006; this study).

Apart from age and gender, results of studies on F^−^ in birds may be influenced by the types of bones from which samples are taken. For example, it has been shown that in the spinal and cranial bones, F^−^ concentrations are higher than in the long bones, and in the compact bone, F^−^ levels are higher than in spongy bone (Taylor and Kirkley 1967, 1960; Xie and Sun 2003). Also in the weaning rat control group, F^−^ concentration in the ash of vertebra was from 10 to 50 % higher than in the femur, with the greatest differences in the youngest groups of animals (Dunipace et al. 1995).

### F^−^ in the brain and pineal gland

In general, it is believed that the mammalian blood–brain barrier is relatively impermeable to F^−^, yet it does not pose an absolute barrier and fluoride has the ability to enter the brain (Spittle 1994; Luke 1997; Shivarajashankara and Shivashankara 2012). Recently, it has been shown that chronic exposure to F^−^ leads to damage of the blood–brain barrier in the spinal cord of rats (Shen et al. 2012). Birds have an incomplete blood–brain barrier, and thus, greater penetration of various substances affecting the central nervous system is possible (Kuenzel et al. 1997).

Despite the increasing frequency of research on the effects of F^−^ on brain tissue at subcellular and biochemical levels, there are very few papers on F^−^ in brain and cerebrospinal fluid (CSF) of vertebrates (including humans). In healthy humans, brain F^−^ concentration ranges from 0.40 to 0.68 mg/kg ww, with levels ≥1.60 or ≥1.80 mg/kg indicating intoxication (Call et al. 1965; Cordero et al. 2004). For people with no identified or diagnosed fluorosis, F^−^ concentrations in CSF and blood differed slightly and amounted to an average of 0.17 and 0.20 mg/l, respectively (Hu and Wu 1988).

The greatest F^−^ concentration in the human brain was detected in a patient after accidental dermal exposure to hydrofluoric acid; the greatest F^−^ in the CSF has been found in a patient who swallowed about 55 g of NaF in a suicide attempt. The corresponding values were 20.4 mg/kg ww (>100 mg/kg dw) and 37 mg/l (Martinez et al. 2007). In the brains of laboratory rodents (rat and mouse) serving as control groups in experiments, F^−^ concentrations ranged from 0.05 to 0.61 mg/kg ww (or from about 0.25 to 3.0 mg/kg dw), while in intoxicated animals, it may reach >5 mg/kg ww or >25 mg/kg dw (Mullenix et al. 1995; Vani and Reddy 2000; Inkielewicz and Krechniak 2003; Tsunoda et al. 2005; Reddy et al. 2011; Lubkowska et al. 2012).

So far, the highest concentration of F^−^ in the brain among vertebrates has been observed in fish kept for 90 days in tanks of water containing ~120 mg/l at almost 590 mg/kg ww (~2,950 mg/kg dw), the bioconcentration factor for nervous tissue (tissue F^−^/water F^−^) was 4.4 (Cao et al. 2014). These facts confirm that F^−^ passes through the blood–brain barrier into the CSF and is deposited in the vertebrate brain at concentrations whose levels depend on the amount of F^−^ intaken with diet and/or water. In the goosander from NW Poland, we detected unexpectedly high concentrations of F^−^ in the brain compared not only to mammalian brains, but also in relation to their concentrations in bone. In the tested individuals, the lowest value in the brain was 86.6 mg/kg dw, almost as much as the highest ever recorded in humans, while the maximum F^−^ observed in the goosander was three times greater (~260 mg/kg dw).

In comparison with the average levels observed in humans and non-intoxicated laboratory rodents, the average F^−^ concentration in the brain of the goosander turned out to be 2–3 orders of magnitude greater. Based on data from Call et al. (1965), we calculated the F^−^ concentration in human bone (501 mg/kg dw in vertebrae) and the brain (1.5 mg/kg dw). In humans, the concentration of F^−^ in bone is >330 times greater than in the brain. However, in our study on goosander, the corresponding rate was only 1.8.

**Fig. 2:**
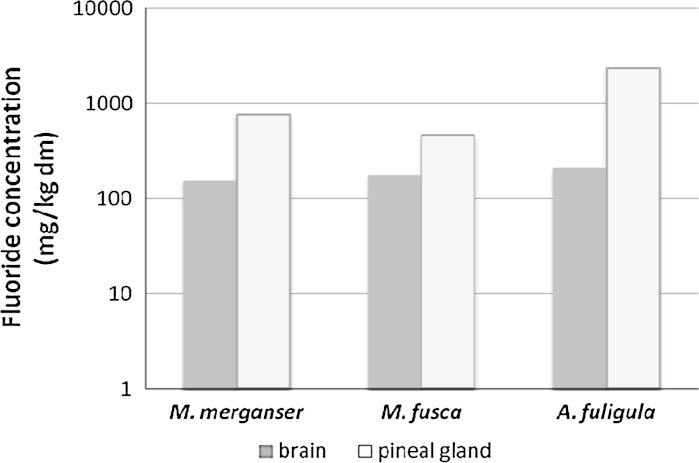
Comparison of median values of F^−^ concentration in the brain and pineal gland of the adult goosander *M. merganser*, velvet scoter *M. fusca* and tufted duck *A. fuligula* from the Odra delta, NW Poland

So far, there has only been one paper on F^−^ in avian brains—on two other species of ducks wintering in the Odra estuary: the velvet scoter *Melanitta fusca* and the tufted duck *Aythya fuligula* (Kalisinska et al. 2012). The former species breeds mainly in the east and north of Europe, including Fennoscandia, and the latter almost across the entire Eurasia, including Poland where it is considered to be a moderately common and partly settled species, especially in the west and northwest of the country (Hagemeijer and Blair 1997; Tomialojc and Stawarczyk 2003). In fall and winter, both species feed primarily on zoobenthic invertebrates, including mollusks such as the zebra mussel *Dreissena polymorpha* (del Hoyo et al. 1992; De Leeuw et al. 1999). This mollusk is very common in the Szczecin Lagoon, although in recent years, there has been a drastic reduction in the abundance of this species in some parts of this water body (Wolnomiejski and Wozniczka 2008; Radziejewska et al. 2009). It is worth noting that the zebra mussel is very resistant to F^−^ in water, and its concentration reaches a higher values in soft tissues than in the calcium carbonate-rich shells, which has been experimentally shown by Del Piero et al. (2012). In the adult velvet scoter and tufted duck, median values of brain F^−^ concentrations were greater than in the tissue of the adult goosanders in this study (150 mg/kg dw) o 16 and 35 %, respectively (Fig. 2).

The causes of such a high concentration of F^−^ in the brain of wild ducks and the potential effects on this most crucial organ are not known. Perhaps the avian blood–brain barrier is more permeable to F^−^ and/or more strongly damaged in birds exposed to F^−^ in the long term than in the rat or other mammals. The goosander, velvet scoter and tufted duck are diving predatory ducks, with high activity and metabolism (especially during flying and diving), feeding on food of aquatic origin, including fish and the zebra mussel which usually contain significant levels of F^−^, but also calcium (Camargo 2003; Del Piero et al. 2012).

In the Odra estuary, daily intake of F^−^ by a goosander weighing about 2 kg is ~15 mg, i.e., 7.5 mg/kg body mass/day. For comparison, in people inhabiting areas with temperate climates, daily exposure to F^−^ is about 0.6 mg/adult/day if the water is not fluoridated (Fawell et al. 2006). Thus, daily intake of F^−^ by monophagous fish-eating goosander was at least ten times greater than among humans with a varied diet. In people exposed to a low intake of fluoride, no changes were observed in the nervous system (Fawell et al. 2006). Moreover, in rats receiving 10 mg NaF/kg body mass daily, i.e., similar to goosander from the Odra estuary, degenerative changes were observed in the cerebellar cortex, part of the encephalon responsible for locomotor coordination (Afifi 2009). Any damage caused by F^−^ in the cerebellum of intensively flying and diving birds which are at the same time long-distance migrants may have very serious consequences for their populations.

Out of the total pool of F^−^ accumulated in the body, the greatest share is found in the bone in which F^−^ is quite persistently bound. However, some F^−^ occurs in the blood and soft tissues, from where it is expelled with feces and, to a lesser extent, with the secretion of salt glands (Culik 1987). It seems that an excess of F^−^ present in the soft tissues, particularly in the brain, from where the body is unable to efficiently remove F^−^, may be permanently or temporarily fixed in the form of biologically inactive deposits. There are no known adaptive mechanisms to prevent or reduce brain F^−^ poisoning in birds. One can only assume the important role of Ca (as in mammals), as Ca^2+^ ions bind to F^−^ to form relatively insoluble salts, such as CaF_2_ (Monsour and Kruger 1985; Tsuchida and Yanagisawa 1985).

In humans, at least some calciferous concretions present in the soft tissues are composed of hydroxyapatite and fluorapatite (Ostrowski et al. 1980). Among the soft tissues, calcium and fluorine compounds in the form of calcifications are noticed mainly in mammal brains. In various parts of the brain, calcified concretions are formed naturally (brain sands, corpora arenacea, or acervuli), with the intrapineal calcifications best described, mainly in mammals, although they have also been detected in birds (Fejer et al. 2001; Makariou and Patsalides 2009). Extrapineal calcifications visible in the choroid plexus of lateral ventricles, meninges, and elsewhere in the habenular commissure, are known as basal ganglia and have been best examined in humans (Koshy and Vettivel 2001; Kiroglu et al. 2010; Uduma et al. 2011). There is no data on extrapineal calcifications in birds in available literature.

Luke (2001) has shown for the first time that F^−^ readily accumulates in the human pineal gland. In her study, intrapineal calcifications in the brains of persons aged about 80 contained on average 9,000 mg F^−^/kg, and the average concentration in the pineal gland was 297 ± 257 (14–875) mg F^−^/kg wm, so about 1,485 ± 1,285 mg F^−^/kg dm. Compared with humans, the pineal gland of the adult goosander had an almost 40 % lower concentration. Among the three species of duck from the Odra estuary, the tufted duck had the greatest median F^−^ concentration in the pineal gland while the velvet scoter had the lowest (Fig. 2). F^−^ concentration in the tufted duck (2,360 mg F^−^/kg dm) was almost three times higher than in the goosander, and as much as five times greater compared with the velvet scoter. The pineal gland-to-brain ratios of F^−^ concentrations in adult goosanders and tufted ducks were 4.9 and 2.7, but in the velvet scoter, it exceeded 11.5. It seems that the greater F^−^ accumulation in the pineal gland and brain of the tufted duck, compared with the goosander and velvet scoter, was caused by the duration of feeding on F^−^ containing material. The tufted duck has breeding and wintering grounds in Poland, where it may live over the entire year (Tomialojc and Stawarczyk 2003). A decisive part of the goosander population and all velvet scoters wintering in the Odra estuary spend their breeding season in the north and northeast of Europe, where waters in lakes and rivers (and so probably fish and invertebrates in those waters) have a few times lower F^−^ concentrations than in Poland. As shown in the study on F^−^ in stream water of European countries, in central and western Poland, F^−^ concentrations are 5–7 times higher than in most of Scandinavia, especially Norway, and range from 0.23 to 0.36 mg F^−^/l (Salminen et al. 2005) compared with Norwegian lakes usually with <0.005 mg F^−^/l (Skjelkvale 1994). Fluoride concentrations, both in stream water of northwestern Poland (the Gunica River flowing into the Odra River near the “Police” chemical plant with 0.36 mg F^−^/l) and tap water in Szczecin (with an average F^−^ level at 0.28 mg/l) are lower than the thresholds given by the World Health Organization, USA Environmental Protection Agency, European Union and Polish drinking water standards at 1.5 or 2.0 mg F^−^/l (Fawell et al. 2006; Lubkowska 2009; Telesinski et al. 2011; EPA 2012).

The reported great differences in brain F^−^ concentrations between birds and mammals, and to a lesser extent in the pineal gland, may result not only from food preferences, population areas and the amount of F^−^ present in the environment, but mainly from their different biology and anatomy, including the structure of the brain and blood–brain barrier. Experimental studies on rodents indicate that F^−^ ions may decrease the capacity to synthesize glucose, alter the metabolism of glucose, and induct glycogen accumulation in the brain and other tissues (Chinoy et al. 2004; Trivedi et al. 2009; Agalakova and Gusev 2012; Shivarajashankara and Shivashankara 2012).

This type of research has not been conducted on birds, but it is known that birds maintain higher plasma glucose than other vertebrates of similar body mass and, in most cases, appear to store comparatively very little glucose intracellularly as glycogen, and instead store it a bird-specific structure located in the spinal cord (the corpus gelatinosum) and in astrocytes (Moller and Kummer 2003; Braun and Sweazea 2008). The avian nervous system utilizes glucose as a metabolic substrate, but little is known about the metabolism of glycogen in the avian brain, including those diving species at risk of oxygen deficiency (Brown and Ransom 2007; Braun and Sweazea 2008).

The avian regulation of plasma glucose level is insensitive to insulin. The level of this hormone depends on the secretion of melatonin produced by the pineal gland, as shown in many mammalian species (Braun and Sweazea 2008; Peschke et al. 2013). A deficiency of this hormone may have very serious consequences at the biochemical level of individuals and also seasonal population migrations. Recently, the pineal gland of the common gull *Larus canus* has been shown to include paracrystalline structures containing large amount of glycogen. These peculiar structures have not been previously described in the pineal organs of birds or in any vertebrate (Przybylska-Gornowicz et al. 2012).

In this context, it seems significant to note that the examined common gull (feeding on fish) came from the Polish Baltic coast in Gdansk. This area is heavily influenced by the industrial emissions of Gdańska Rafinery, Fosfory of Gdańsk (phosphatic fertiliser plant), Gdansk Shipyard, industrial waste stockpiles (in particular phosphogypsum), slag and ash heaps—all emitting many pollutants, including F^−^ (Bombik et al. 2011). It cannot be excluded that environmental conditions and food (terrestrial and aquatic invertebrates, small fish) contaminated by F^−^ contributed to such spectacular glycogen accumulation, although this requires further studies.

Research has shown that even relatively low levels of F^−^ in water may be the cause of significant accumulation in aquatic organisms, and then in the bodies of ducks feeding on invertebrates and fish, including the brain and the pineal gland. There are no known behavioral and environmental consequences of this phenomenon in aquatic birds. In addition, in the Odra estuary, F^−^ is accompanied by other pollutants (including mercury, cadmium, lead, manganese) that accumulate in the bodies of birds wintering there, as shown in previous works (Kalisinska and Szuberla 1996; Kalisinska et al. 2012).

Many metals in mammals interact with F^−^, which may contribute to the increased accumulation of toxic elements and micronutrient deficiency in animals and humans (Singh and Kanwar 1981; Reddy et al. 2009; Sawan et al. 2010; Rocha Gomes Torres et al. 2011). The total negative effect of these trace elements and F^−^ and their interactions in the bodies of birds may be one of the environmental reasons for the drastic decrease in the number of some species around the Szczecin Lagoon, including the goosander and the velvet scoter (Ronka et al. 2005; Lawicki et al. 2008; Wilk et al. 2010; BirdLife International 2013).
